# Metagenomic Sequencing of Diamondback Moth Gut Microbiome Unveils Key Holobiont Adaptations for Herbivory

**DOI:** 10.3389/fmicb.2017.00663

**Published:** 2017-04-26

**Authors:** Xiaofeng Xia, Geoff M. Gurr, Liette Vasseur, Dandan Zheng, Huanzi Zhong, Bingcai Qin, Junhan Lin, Yue Wang, FengQin Song, Yong Li, Hailan Lin, Minsheng You

**Affiliations:** ^1^State Key Laboratory of Ecological Pest Control for Fujian and Taiwan Crops, Fujian Agriculture and Forestry UniversityFuzhou, China; ^2^Institute of Applied Ecology, Fujian Agriculture and Forestry UniversityFuzhou, China; ^3^Key Laboratory of Integrated Pest Management for Fujian-Taiwan Crops, Ministry of AgricultureFuzhou, China; ^4^Fujian-Taiwan Joint Innovation Centre for Ecological Control of Crop Pests, Fujian Agriculture and Forestry UniversityFuzhou, China; ^5^Graham Centre, Charles Sturt UniversityOrange, NSW, Australia; ^6^Department of Biological Sciences, Brock UniversitySt. Catharines, ON, Canada; ^7^BGI-ShenzhenShenzhen, China

**Keywords:** diamondback moth, bacteria diversity, gut symbiosis, carbohydrate biodegradation, plant defense, phenolic compounds, amino acids, reactive oxygen species

## Abstract

Herbivore specialists adapt to feed on a specific group of host plants by evolving various mechanisms to respond to plant defenses. Insects also possess complex gut microbiotas but their potential role in adaptation is poorly understood. Our previous study of the genome of diamondback moth, *Plutella xylostella*, revealed an intrinsic capacity to detoxify plant defense compounds, which is an important factor in its success as a pest. Here we expand on that work with a complete taxonomic and functional profile of the *P. xylostella* gut microbiota obtained by metagenomic sequencing. Gene enrichment in the metagenome, accompanied by functional identification, revealed an important role of specific gut bacteria in the breakdown of plant cell walls, detoxification of plant phenolics, and synthesis of amino acids. Microbes participating in these pathways mainly belonged to three highly abundant bacteria: *Enterobacter cloacae, Enterobacter asburiae*, and *Carnobacterium maltaromaticum*. Results show that while the gut microbial community may be complex, a small number of functionally active species can be disproportionally important. The presence of specific enzymes in the microbiota community, such as supporting amino acid synthesis, digestion and detoxification functions, demonstrates the beneficial interactions between *P. xylostella* and its gut microbiota. These interactions can be potential targets for manipulation to provide novel pest management approaches.

## Introduction

The eukaryote gut co-evolves with a microbiome that plays important roles in digestion, nutrition, development, and immune responses (Warnecke et al., 2007; Dong et al., 2009; Engel et al., 2012; Engel and Moran, 2013b). Animals are usually deficient in genes necessary for some key metabolic functions, such as the synthesis of essential amino acids and degradation of cellulose (Ley et al., 2008; Hansen and Moran, 2011; Zhu et al., 2011). These functions can be accomplished by their symbionts; such as the case for cellulose breakdown by the termite (*Nasutitermes*) gut bacteria (Warnecke et al., 2007), vitamin B12 biosynthesis and pectin degradation by honey bee (*Apis mellifera*) gut bacteria (Engel et al., 2012; Engel and Moran, 2013a), the production of amino acids by the pea aphid (*Acyrthosiphon pisum*) symbiont *Buchnera aphidicola* (Hansen and Moran, 2011), and fenitrothion insecticide degradation by the bean bug (*Riptortus pedestris*) symbiont *Burkholderia* (Kikuchi et al., 2012). The significance of these microbes within the gut has prompted the intestinal microbiota to be considered a separate organ of the host (Possemiers et al., 2011). Others have proposed that an animal with its microbiota forms a holobiont, which is subject to common selection pressures (Zilber-Rosenberg and Rosenberg, 2008).

The diamondback moth, *Plutella xylostella* (L.) (Lepidoptera: Plutellidae), is one of the world's most destructive pests that has co-evolved with the cruciferous plants, such as cabbage, broccoli, and cauliflower (Talekar and Shelton, 1993). This lepidopteran is difficult to control because it has developed resistance to most classes of insecticides (Baxter et al., 2005; Zalucki et al., 2012). Many studies have focused on the bacterial diversity of the *P. xylostella* gut based on culturing and denaturing gradient gel electrophoresis (DGGE) methods (Indiragandhi et al., 2007; Lin et al., 2015a,b) but these only partially reveal the gut microbial diversity. Our previous study based on the sequencing of V6 region of 16S rRNA supplied a more complete understanding of the *P. xylostella* gut microbiota than earlier culturing-based studies (Xia et al., 2013). Yet a full understanding of bacterial community structure coupled with functional studies has been lacking.

Metagenomics is a powerful tool for comprehensive characterization of the gut microbial diversity as it avoids the need for culturing (Tringe et al., 2005). Studies based on metagenomics can provide full taxonomic profiles of the holobiont. The present study extended our previous research on the *P. xylostella* genome (You et al., 2013) by examining the hologenome, providing insights into the co-evolution of this herbivore's gut microbiota. In this work, we also have complemented the hologenome with functional information that underpins our understanding on the key holobiont adaptations for herbivory. We revealed that, despite the complexity of this microbiome, a small number of bacterial species of particular importance to key digestive and detoxification processes underpin the success of this globally significant pest.

## Materials and methods

### Insect rearing

A susceptible *P. xylostella* strain (hereafter, SS) was collected in July 2004 from a vegetable field in Fuzhou (26.08°N, 119.28°E), Fujian province, south eastern China. All necessary permits were obtained from the Institute of Plant Protection of the Fujian Academy of Agricultural Sciences. The larvae were fed on radish seedlings at 25 ± 2°C, 70–80% relative humidity and a 16 h light/8 h dark photoperiod without exposure to insecticides. Pupae were collected and transferred to a 500 mL plastic bottle where emerging adults were fed 10% honey solution to mate and lay eggs. Neonates were able to pass through perforations in these bottles and drop onto radish plants below.

### Collecting *P. xylostella* gut contents and DNA extraction

To collect the gut contents, 200 individuals each of 3rd-instar larvae, pupae, and adults were randomly sampled regardless of sex. The samples were surface-sterilized with 75% ethanol for 90 s and rinsed with sterilized-deionized water. After dissection, the gut contents were homogenized with 1 ml sterile deionized water and frozen at −80°C for DNA extraction. Total DNA from the larvae, pupae and adults samples was extracted using the QIAamp® DNA Stool Mini Kit (Gene Company Limited, Lee Chung Street, Hong Kong, P.R.C.) following the manufacturer's protocol. The DNA products were run on 1.0% agarose gels and recovered for library construction and sequencing.

### DNA library construction and sequencing

DNA library construction was performed following the manufacturer's instructions (Illumina, San Diego, California, U.S.). We constructed one paired-end (PE) library with insert size of 350 bp for each life stage sample. Then, high-throughput sequencing was performed using Illumina Hiseq 2,000 to obtain the metagenome data. The read length for each PE end was 100 bp. The raw reads that contained three or more “N” or adapter contamination were discarded. If there were continuous low-quality (Phred quality score <20) bases at the end of one read, these bases were trimmed from the read. The trimmed reads with lengths below 30 bp were discarded, and host contaminated reads were removed; the remaining effective reads were used for further analysis. For each sample, the effective reads were assembled to obtain long contigs by using SOAP *de novo* assembler (Li et al., 2010). Then, the contigs longer than 500 bp were used to predict ORFs by the Meta Gene program (Noguchi et al., 2006). Using ORFs longer than 100 bp from all samples, we constructed a non-redundant gene catalog and effective reads from each sample were aligned against it by SOAP2 (Li et al., 2009) using the criterion ≥90% identity. We translated the aligned results to gene relative abundance by counting the number of reads that mapped to the gene. Each gene in the gene catalog was aligned against NCBI NR database for taxonomy classification and against KEGG database for functional annotation by the BLASTP program with the criterion of expect value <10^−5^. Based on the annotation information of the gene catalog, we summed the relative abundances of the genes from the same taxon or same KO (KEGG ortholog group) to generate the taxonomic profile or KO functional profile. The raw sequences of gut microbiota of *P. xylostella* larvae, pupae, and adults were deposited to the SRA (Sequence Read Archive) database under accession number of SRP018285.

### Biomass degradation experiments

Fifteen 4th-instar larvae were randomly sampled regardless of sex. The samples were surface-sterilized with 75% ethanol for 90 s and rinsed with sterilized-deionized water. After dissection with a sterilized scalpel, the gut contents were homogenized with 1 mL sterile deionized water. The gut content suspension was diluted to 10^−4^, 10^−5^, and 10^−6^, and 50 μL of the suspension was spotted onto the caboxymethyl cellulose (CMC), xylan, and pectin degrading enzyme isolation medium. The media for CMC, xylan, and pectin degrading enzyme isolations were: CPS1 (g/L): CMC-Na 10 g, peptone 1 g, (NH_4_)_2_SO_4_ 4 g, NaCl 1 g, K_2_HPO_4_ 2 g, MgSO_4_·7H_2_O 0.4 g, agar 20 g, pH 7.0; XPS1 (g/L): xylan 8 g, yeast powder 1 g, (NH_4_)_2_SO_4_ 4 g, K_2_HPO_4_ 2 g, NaCl 0.5 g, MgSO_4_·7H_2_O 0.5 g, agar 20 g, pH 7.0, and PPS1 (g/L): pectin 10 g, yeast powder 1 g, (NH_4_)_2_SO_4_ 4 g, K_2_HPO_4_ 1 g, KCl 0.5 g, MgSO_4_·7H_2_O 0.4 g, FeSO_4_ 0.01 g, agar 20 g, pH 7.0. The plates were cultured at 30°C for 3 days. Bacteria grown on the plates were purified and identified by another identification medium [CPS2 (g/L): CMC-Na 5 g, (NH_4_)_2_SO_4_ 2 g, NaCl 1 g, K_2_HPO_4_ 2 g, MgSO_4_·7H_2_O 0.2 g, CaCl_2_ 0.1 g, agar 20 g, pH 7.0; XPS2 (g/L): xylan 5 g, (NH_4_)_2_SO_4_ 2 g, K_2_HPO_4_ 2 g, NaCl 1 g, MgSO_4_·7H_2_O 0.2 g, agar 20 g, pH 7.0, and; PPS2 (g/L): pectin 5 g, (NH_4_)_2_SO_4_ 2 g, K_2_HPO_4_ 1 g, NaNO_3_ 3 g, KCl 0.5 g, MgSO_4_·7H_2_O 0.2 g, CaCl_2_ 0.15 g, FeSO_4_ 0.01 g, agar 20 g, pH 7.0], which contained less substrate than the isolation medium. The potentially degrading bacteria grown on the plates (CPS2, XPS2, and PPS2) at 30°C for 7 days were used for enzyme activity identification. The CPS2 plates were dyed with 0.1% Congo red for 10 min, the plates were floated with 1 mol/L NaCl for 5 min, and CMC degrading enzyme activity was determined by the visible clearance zones. The XPS2 and PPS2 plates were dyed by 0.1% Lugel solution and Bromophenol blue for 5 min, respectively, to see whether clearance zones became visible.

### Bacteria identification

The representative bacteria that contained cellulose, xylan and pectin degrading activities were purified and named as DBMG1, DBMG2, DBMG3, DBMG4, DBMG5, and DBMG6. Strains were cultured in LB medium (g/L): peptone 10 g, yeast powder 5 g, NaCl 10 g, pH 7.0. Genomic DNA of each strain was isolated by Rapid Bacterial Genomic DNA Isolation Kit (Sango biotech, China). To amplify 16S rRNA of each strain for bacterial identification, universal primers targeting the 16S rRNA, 27F (5′-AGAGTTTGATCCTGGCTCAG-3′) and 1492R (5′-GGTTACCTTGTTACGACTT-3′) were designed as previously described (Weisburg et al., 1991). The PCR was carried out in a total volume of 15 μL: H_2_O 10.5 μL, 10 × PCR ExTaq Buffer 1.5 μL, DNA template (100 ng/μL) 1.0 μL, 27F (10 mmol/L) 0.5 μL, 1492R (10 mmol/L) 0.5 μL, dNTP 1.0 μL, ExTaq (5 U/μL) 0.15 μL. After initial denaturation at 95°C for 5 min, amplification was performed using 30 cycles of 30 s at 95°C, 30 s at 56°C, 60 s at 72°C, followed by a final extension at 72°C for 10 min. Amplification products were then run on 1.0% agarose gels and purified, and the products were sequenced by ABI PRISM 3730. The 16S rRNA sequences were submitted to the NCBI GenBank (GenBank Accession Numbers: KT957438, KT957439, KT957440, KT957441, KT957442, and KT957443 which corresponding to the above strains of DBMG1, DBMG2, DBMG3, DBMG4, DBMG5, and DBMG6, respectively).

### Phylogenetic analysis

The 16S rRNA sequences of DBMG1, DBMG2, DBMG3, DBMG4, DBMG5, and DBMG6 were used as queries to do homolog search using the NCBI blast program. The homolog sequences were downloaded and aligned by clustalX1.83 with the default settings, phylogenetic analysis was performed by MEGA5.0 based on the Neighbor-Joining method, and 1,000 bootstrap replications were used to assess the phylogenetic tree.

### Phenol biodegradation experiments

The *P. xylostella* gut microbiota were cultured at 30°C in sterilized mineral salt medium (MSM) (g/L): (NH_4_)_2_SO_4_, 0.4; K_2_HPO_4_, 0.4; KH_2_PO_4_, 0.2; MgSO_4_, 0.1; NaCl, 0.1; Fe_2_(SO_4_).H_2_O, 0.01; MnSO_4_.H_2_O, 0.01; for a 100 mg/L final concentration of filter-sterilized phenol as a carbon source, pH 7.5. The 100 mL phenol medium in a 250 mL conical flask with the bacteria was incubated in a rotary shaker at 150 rpm. Phenol degradation was monitored in the colorimetric assay according to established method (Ahmad et al., 2012). Phenol concentration was measured every 12 h for the total 24 h. The experiments were performed in triplicate. Data were analyzed by one-way ANOVA followed by LSD *post-hoc* test using IBM SPSS Statistics 19.

## Results and discussion

### Compositional diversity of the *P. xylostella* gut microbiota

**Figure 1:**
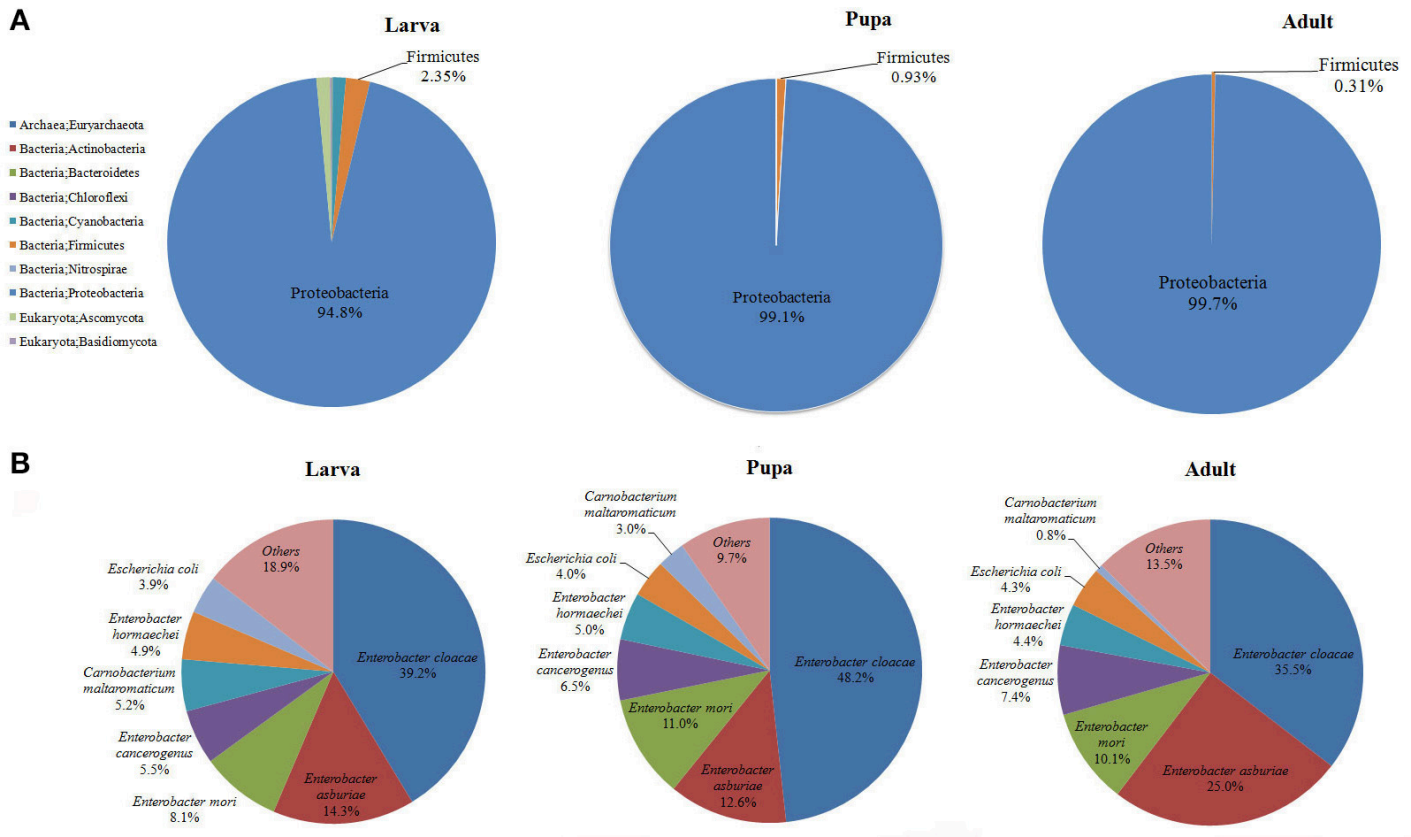
**Proportional composition of microbiota in the ***P. xylostella*** gut generated from the metagenomics data**. Composition at the phylum level **(A)**, and composition at the species level **(B)**.

Gut microbiota of *P. xylostella* larvae, pupae, and adults were sequenced. *De novo* assembly of 3.46 Gb effective reads resulted in 30,282 contigs, and 50,724 ORFs were predicted from the contigs, with a total length of 34.18 Mb (Supplementary Tables 1–3). Taxonomic profiling of the metagenome indicated that the *P. xylostella* gut microbiota was dominated by Proteobacteria (comprising 94.8, 99.1, and 99.7% in the gut of larva, pupa, and adult, respectively), followed by Firmicutes (2.35, 0.93, and 0.31% in larva, pupa, and adult), and to lesser extent, Cyanobacteria, Bacteroidetes, Actinobacteria, Nitrospirae, and the archaea species Euryarchaeota (Figure 1A, Supplementary Table 4). The gut microbiota also contained a relatively small proportion of Eukaryota of the species of Basidiomycota (0.18% in larva, 0.0004% in pupa, and 0.001% in adult) and Ascomycota (1.31% in larva, 0.002% in pupa, and 0.01% in adult). Bacteria represented ~98% of the entire microbiota (Figure 1A, Supplementary Table 4).

The present metagenomic study provided a complete taxonomic profile of the *P. xylostella* gut microbiota (Figure 1, Supplementary Figures 1–5). Overall, 10 phyla and 148 species of microorganisms were identified (Supplementary Table 4). The species diversity is consistent with our previous study of larvae, with 150 OTUs identified at the order level of the *P. xylostella* midgut microbiota based on the V6 region sequencing (Xia et al., 2013). The Enterobacteriales from Proteobacteria (88.5% in larva, 98.7% in pupa, and 99.0% in adult) dominated at the order level followed by Lactobacillales from Firmicutes (1.68% in larva, 0.85% in pupa, and 0.27% in adult). The most abundant species in Enterobacteriales were *E. cloacae* (39.2, 48.2, and 35.5% in larva, pupa, and adult, respectively) and *E. asburiae* (14.3, 12.6, and 25.0% in larva, pupa, and adult; Figure 1B, Supplementary Table 4). Although, the Proteobacteria were dominated by *E. cloacae* and *E. asburiae*, this phylum was represented in the microbiome by 100 species (Supplementary Table 4).

The comparison among the three developmental stages indicated that adults and pupa had similar diversity of gut microbiota, which was lower than the microbiota of larva (Figure 1, Supplementary Figures 1–5). The greater diversity of microbes in the larva compared with the pupa/adult may be the result of differences in the usual food sources among these life stages (plant tissue for larva vs. simple carbohydrates for the adults) and the need of larva to metabolize structurally complex carbohydrates and plant allelochemicals. Previous study based on bacterial culture and PCR-DGGE analysis also suggest that the abundance and diversity of bacteria in the *P. xylostella* gut are much higher in larva than that in pupa/adult (Lin et al., 2015b). Proteobacteria however were more abundant in the pupa and adult than in larva, corresponding with lower abundance of Firmicutes. At the species level, the abundance of *E. asburiae* was greater in the adult stage while *E. cloacae* was more abundant at the larval stage (Figure 1B). *Anopheles gambiae* also shows variation in gut bacterial communities with larva and pupa being similar but different from adults reflecting the change from detritus to blood feeding (Wang et al., 2011).

**Figure 2:**
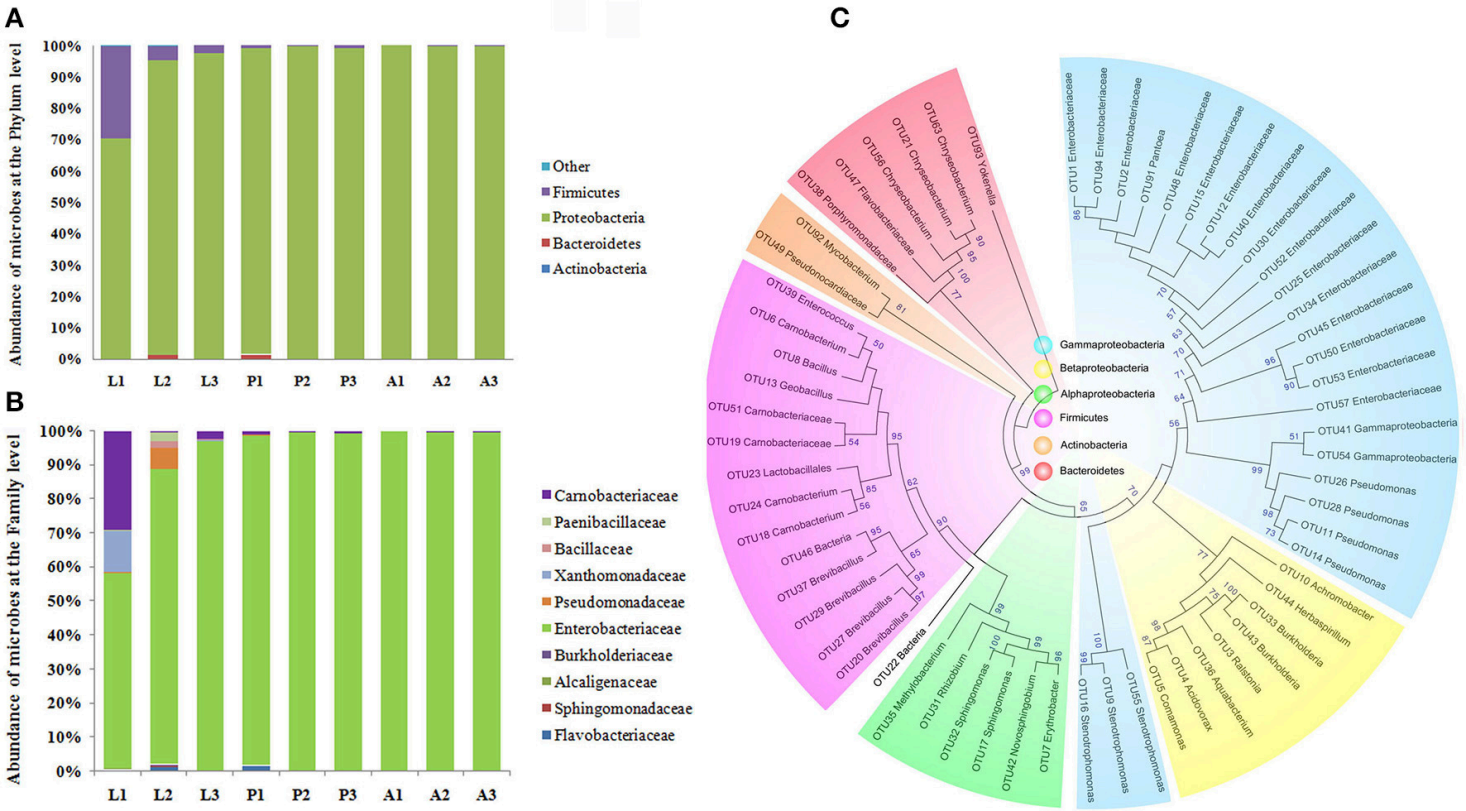
**Proportional composition of microbiota in the ***P. xylostella*** gut generated from the 454 pyrosequencing data**. Composition at the phylum level **(A)**, and composition at the family level **(B)**, Neighbor-Joining method phylogeny of OTUs **(C)**. The phylogenetic tree revealed that the microbiota of *P. xylostella* gut were divided into six clusters corresponding with the phylum of Proteobacteria (Gammaproteobacteria was shown in blue, Betaproteobacteria with light yellow, and the alphaproteobacteria with green), Firmicutes (shown in purple), Actinobacteria (shown in dark yellow), and Bacteroidetes (shown in pink). Most of the sequences from the *P. xylostella* gut fell into the clusters of Proteobacteria and Firmicutes. Bootstrap values >50 were shown in the tree.

In order to validate the results of the composition of *P. xylostella* gut microbiota, a bar-coded pyrosequencing on the V3~V5 of 16S rRNA region in different *P. xylostella* developmental stages of the gut microbiota was carried out (see Supplementary Materials and Methods). A total of 47,872 effective reads from 9 samples with an average of 5,319 reads per sample were produced (Supplementary Table 5). Sixty-two OTUs were calculated based on the 97% cut-off (Supplementary Table 6), which was much less than the metagenomic sequencing and previous sequencing based on the V6 region by Illumina. The rarefaction curves suggested that the pupa and adult reached a plateau. However, the larva was far from reaching the plateau, which might be a reason of the low abundance of OTUs (Supplementary Figure 6). Alpha diversity analysis indicated that the composition of larval gut microbiota was much more complex than that of pupa and adult (Supplementary Table 7). Although, the OTUs were much fewer than in the metagenomics analysis, the main composition and structure were very similar with the metagenomics results. The phylum of Proteobacteria was the dominant taxon in the *P. xylostella* gut microbiota (from 70.42 to 97.44% in larva, 97.44 to 99.58% in pupa, and 99.64 to 100% in adult), followed by Firmicutes (from 2.45 to 29.48% in larva, 0.42 to 1% in pupa, and 0 to 0.36% in adult; Figure 2). More than 99% of long reads based on the bar-coded pyrosequencing could be identified to the family level (Supplementary Table 8). The Enterobacteriaceae (Phylum: Proteobacteria, Order: Enterobacteriales) dominated the gut ecology (from 57.89 to 97.29% in larva, 97.33 to 99.58% in pupa, and 99.61 to 99.86% in adult). Carnobacteriaceae (Phylum: Firmicutes, Order: Lactobacillales) was the second most abundant family (from 0.42 to 29.12% in larva, 0.42 to 1% in pupa, and 0 to 0.36% in adult; Supplementary Table 6). These results were consistent with the metagenomic analysis.

Results showing dominance of Proteobacteria and to a lower extend Firmicutes were consistent among the three methods we used in the study. Yun et al. (2014) report in a study of 218 insect species, a similar pattern where Proteobacteria and Firmicutes represent 82.8% of the total sequences. However, Lactobacillales and Vibrionales (Phylum Proteobacteria) were 10 times more abundant in our previous study based on the sequencing of V6 region of 16S rRNA (Xia et al., 2013) than the other two methods. Variation in abundance among the different sequencing methods may be due to differences in developmental stages and physiological conditions of the individuals (Yun et al., 2014; Mandal et al., 2015). The low abundance of Vibrionales detected in metagenomics, and even not detected by 454 pyrosequencing may be due to a very low abundance in *P. xylostella* gut environment compared to its high abundance in the previous study (Xia et al., 2013) caused by the enrichment of this order by the V6 amplification. It is possible that insect collection in different seasons or physiological health of the samples may result in different gut ecology (Wong et al., 2011; Yun et al., 2014). The method of bacterial DNA extraction also may have affected the structure of gut symbiont (Wagner Mackenzie et al., 2015), different sequencing strategies targeted different genes or different regions can also affect the result (Sun et al., 2013; Filippidou et al., 2015). In summary, there is a need to examine which research methods give more objective and real evaluation of gut microbial diversity.

Comparative analyses of the diversity of different gut microbiota have revealed that different insects host different gut microbiota. The honey bee midgut is dominated by Proteobacteria and Actinobacteria, and *Snodgrassella* is important (Engel et al., 2012). Termites contain Proteobacteria, Spirochaetes, and Fibrobacteres, the latter of which functioned in cellulose digestion (Hongoh et al., 2006; Warnecke et al., 2007), while the mosquito gut microbiome is dominated by Proteobacteria and Cyanobacteria (Wang et al., 2011). The environmental habitat, phylogeny of host, diet, and developmental stages all determined the insect gut bacterial diversity (Yun et al., 2014). Our finding underlined the importance of gut microbiota identity and composition in the evolutionary adaptation of insects to differing diets including difficult-to-digest and well-defended plant material (Hongoh et al., 2005).

### Functional diversity of the *P. xylostella* gut microbiota

**Figure 3:**
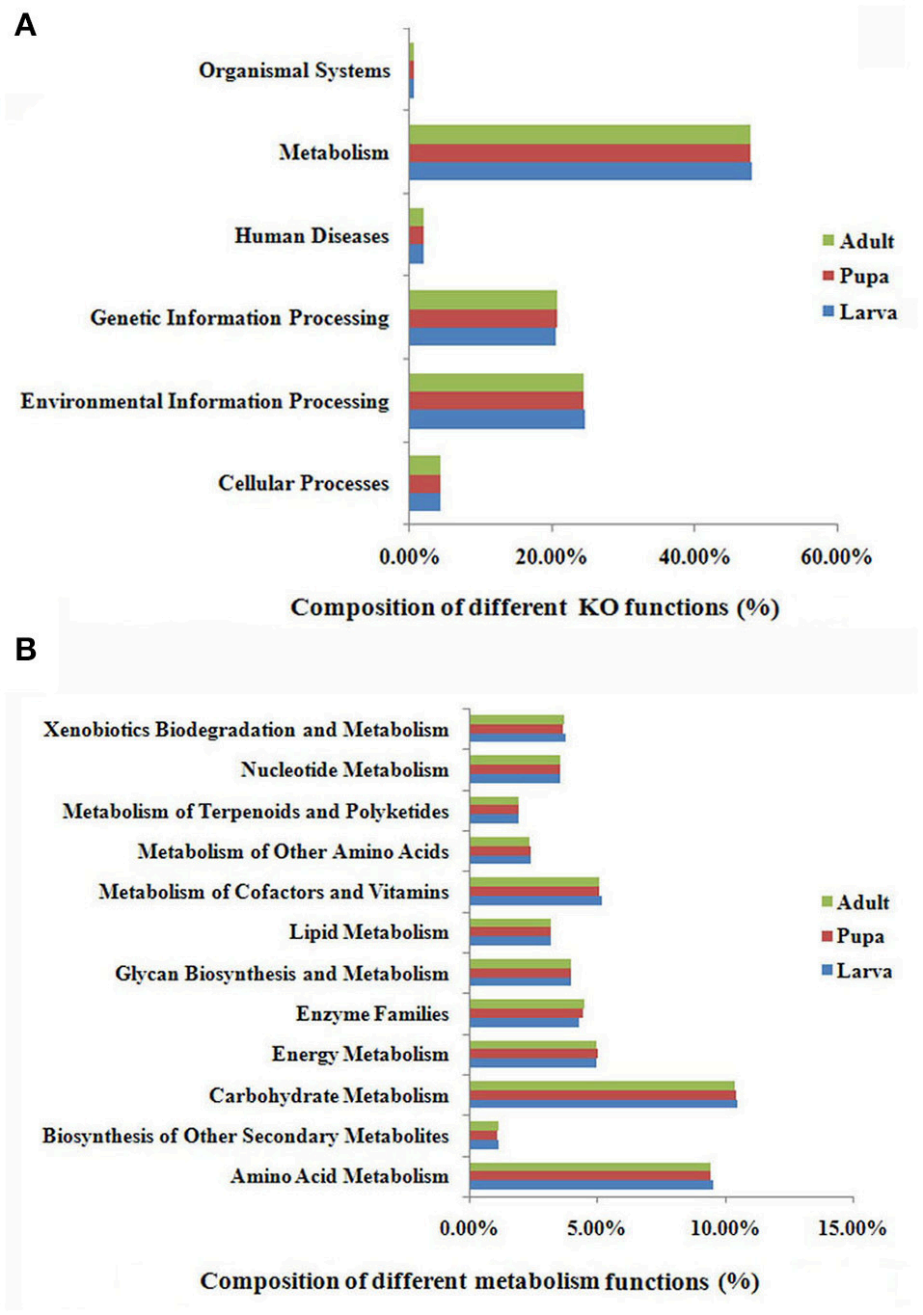
**KEGG ortholog (KO) group annotation of the ***P. xylostella*** gut microbiota metagenome**. Functional composition of the gut microbiota metagenome **(A)**, and function of metabolism of the gut microbiota metagenome, the “Enzyme Families” in the figure represent the enzymes of “Peptidases” and “Protein kinases” that were detected in the DBM gut metagenome **(B)**.

MetaGene analysis revealed 15,046, 18,324, and 18,197 non-redundant protein-coding genes in the gut microbiota of the *P. xylostella* larva, pupa, and adult, respectively (Supplementary Table 3). The gene functions annotated by the Kyoto Encyclopedia of Genes and Genomes (KEGG) pathways indicated that metabolic function was the most abundant in the metagenome (representing 48.1, 47.9, and 47.9% of the total KO function in gut microbiota of the *P. xylostella* larva, pupa, and adult, respectively; Figure 3A). Further analysis showed the participation of gut bacteria in metabolic activities associated with glycans, carbohydrates, amino acids, vitamins, xenobiotics, and terpenoids, which are linked to digestion, nutrition, and detoxification (Figure 3B). The most enriched functions within these activities were carbohydrate metabolism (representing 10.5, 10.4, and 10.4% of the total KO function in larva, pupa, and adult, respectively) and amino acid metabolism (9.6% in larva, 9.4% in pupa, and 9.4%, in adult; Figure 3B). This indicated that gut microbiota might be involved in food digestion and nutrition. The *Anoplophora glabripennis* metagenome also shows enriched pathways involved in the metabolism of carbohydrates and amino acids, which play key roles on woody tissue digestion and nutrient synthesis (Scully et al., 2013). Interestingly, the different categories of KEGG ortholog in larva, pupa, and adult were very similar. This could be due to the large abundance of Proteobacteria in all developmental stages.

Brassicaceae, the exclusive food of *P. xylostella*, contains defense compounds such as glucosinolates, which are a deterrent for most insect species (Ratzka et al., 2002). *P. xylostella* has adapted to overcome the toxicity of these compounds (You et al., 2013). Xenobiotic biodegradation (representing 3.8, 3.7, and 3.7% of the total KO functions in larva, pupa, and adult, respectively) and terpenoid metabolism (2.0% in larva, and 1.9 in both pupa and adult; Figure 3B) suggested that the gut microbiota had adapted to the heavily defended diet of *P. xylostella*. KEGG ipath2 analysis showed that the gut microbiota metagenome exhibited more metabolic pathways than the *P. xylostella* genome with 18,071 genes (You et al., 2013; Supplementary Figure 7), potentially providing the host insect with advantages in digestion, nutrition, and overcoming toxicity of plant defense compounds.

### Carbohydrate breakdown activity of the *P. xylostella* gut microbiota

The enzymatic degradation of cellulose and hemicellulose by animals can be difficult so its presence in the diet hinders digestion (Morrison et al., 2009; Suen et al., 2010; Zhu et al., 2011). Previous studies have revealed that some microbes possess a subset of enzymatic systems for hydrolysis of plant biomass (Morrison et al., 2009; Suen et al., 2010; Hess et al., 2011). Based on the Carbohydrate-Active Enzymes (CAZy) database (http://www.cazy.org; Lombard et al., 2014), a total of 2,469 genes from six families, which contained modules for catalyzing the breakdown or modification of carbohydrates and glycoconjugates, were detected in the *P. xylostella* gut microbiota. These were glycoside hydrolases (GHs), glycosyltransferases (GTs), polysaccharide lyases (PLs), carbohydrate esterases (CEs), auxiliary activities (AAs), and carbohydrate binding modules (CBMs) (Supplementary Tables 9, 10). GHs are a widespread group of enzymes important in the degradation of plant cell wall that hydrolyse the glycosidic bond between two or more carbohydrates or between a carbohydrate and a non-carbohydrate moiety (Henrissat and Davies, 1997). Fifty-five different GH families were found in the *P. xylostella* metagenome. Among them, GH5 is linked to the activity of endoglucanase, and GH9 to the activities of endoglucanase and β-glucosidase (Lombard et al., 2014). The *P. xylostella* gut microbiota lacked debranching enzymes but had abundant oligosaccharide-degrading enzymes (Supplementary Tables 9, 10).

Comparative analysis of the profiles of GH families detected from the metagenomes of the termite hindgut (Warnecke et al., 2007), honey bee gut (Engel et al., 2012), beetle (*A. glabripennis*) gut (Scully et al., 2013), leaf-cutter ant garden (Suen et al., 2010), and panda gut (Zhu et al., 2011) revealed that GH5 cellulases were most abundant in the termite hindgut and the least in guts of honey bee and panda (Supplementary Table 10). Honey bees consume only pollen and do not require powerful cellulose degradation (Engel and Moran, 2013a). Though bamboo is rich in cellulose, the panda has a very low digestion efficiency, reflecting the low abundance and diversity of cellulose and hemicellulose digestion enzymes in its metagenome (Zhu et al., 2011). In contrast, cellulases were much more diverse in wood-feeding termites and *A. glabripennis* where endohemicellulases and debranching enzymes were also diverse and highly abundant. The high abundance and diversity of biomass digestion enzymes in the gut microbiota of these two species not only suggest their function in plant digestion but also indicate the possible co-evolution of the insect gut microbiome with diet (Warnecke et al., 2007; Scully et al., 2013). The overall abundance and diversity of the cellulases and endohemicellulases in the *P. xylostella* gut microbiota were at intermediate levels compared to those found in termites and honey bees (Supplementary Table 10). The Brassicaceae, exclusive food of *P. xylostella*, contains over 3,000 species (Hall et al., 2002) and is rich in cellulose and hemicellulose in cell walls. The high abundance of GH5 cellulases in the *P. xylostella* gut microbiota may contribute to its adaptive capacity to feed on these plants, specifically to maximize the extent to which these complex substrates are digested.

**Figure 4:**
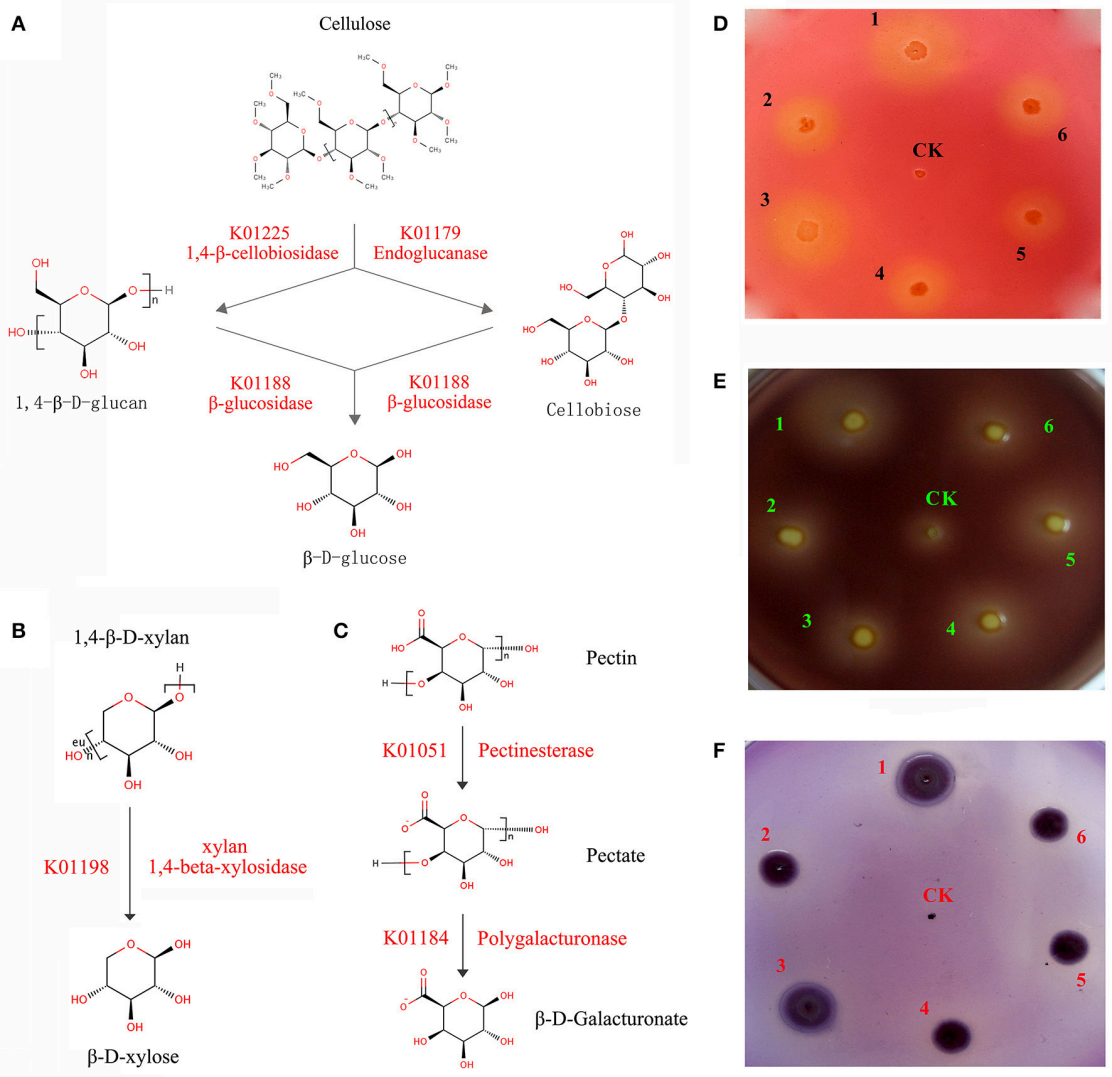
*****P. xylostella*** gut bacteria-mediated metabolism of biomass**. The schematic of biodegradation of caboxymethyl cellulose (CMC) **(A)**, xylan **(B)**, and pectin **(C)** with red words beside the arrow lines representing the degradation enzymes identified from the metagenome, and experimentally validated degradation of CMC **(D)**, pectin **(E)**, and xylan **(F)** with *Escherichia coli* used as negative control (CK). CMC, pectin and xylan degradation is detected by the formation of clearance zones around bacterial lawns, respectively, the *Escherichia coli* as negative control was cultured in the center of plates. The numbers of 1–6 in **(D–F)** represented that the six strains (DBMG1, DBMG2, DBMG3, DBMG4, DBMG5, and DBMG6) of CMC-, xylan-, and pectin-biodegrading bacteria isolated from *P. xylostella* gut. All these six strains were subjected to the genus of *Enterobacter* spp. The GenBank Accession Numbers: KT957438, KT957439, KT957440, KT957441, KT957442, and KT957443 were corresponding to the above strains of DBMG1, DBMG2, DBMG3, DBMG4, DBMG5, and DBMG6, respectively. The clearance zones in the plates **(D–F)** produced by the six strains indicated that the ability of biomass degrading.

A series of enzymes associated with cellulose and hemicellulose hydrolysis was found to be encoded by *C. maltaromaticum* and *Enterobacter* living in the *P. xylostella* gut, including 1,4-beta-cellobiosidase, endoglucanase, and beta-glucosidase. Involved in xylan hydrolysis, 1,4-beta-xylosidase was encoded by *Enterobacter*, whilst pectinesterase and polygalacturonase for pectin biodegradation were encoded by both *C. maltaromaticum* and *Enterobacter* (Figures 4A–C, Supplementary Table 11). According to *P. xylostella* genomic data (You et al., 2013), none of these enzymes are encoded by the host insect genome. KEGG analysis also suggested the capacity of the *P. xylostella* gut microbiota to influence the starch and sucrose metabolic pathways (Supplementary Figure 8). These enzymes were widely involved in the metabolism of carbohydrate, which indicated their potential roles in the digestion of Brassicaceae tissue.

The ability of the six strains (DBMG1, DBMG2, DBMG3, DBMG4, DBMG5, and DBMG6) isolated from *P. xylostella* gut to degrade CMC, xylan, and pectin was confirmed by the formation of clearance zones around bacterial lawns (Figures 4D–F). The CMC is a cellulose derivative obtained by chemical modification of natural cellulose, the degradation ability of CMC indicated the activity of endoglucanase, which would be important for carbohydrate degradation, even cellulolytic activity (Hägerdal et al., 1978). The six strains were identified as genus *Enterobacter* by whole-length 16S rRNA and phylogenetic analysis (Supplementary Figure 9). The phylogenetic tree suggested that the six *Enterobacter* spp. exhibited different 16S rRNA identities and performed different biodegradation activities (Figures 4D–F, Supplementary Table 12). DBMG1 and DBMG3 formed an independent clade, and were more effective at biodegradation, especially for CMC and xylan, than the other clade containing DBMG2, DBMG4, DBMG5, and DBMG6 (Figures 4D–F, Supplementary Figure 9). In honey bee, genetic variation among different species of bacteria may reflect functional divergence within its gut (Engel et al., 2012). Our results suggest that within the *P. xylostella* gut, the most abundant species (*Enterobacter* spp.) contribute to plant cell wall degradation. This capacity enhances food utilization efficiency for the insect host (Warnecke et al., 2007).

### Plant defense compound detoxification activity in the gut microbiota of *P. xylostella*

Herbivory requires the evolution of a broad range of mechanisms to not only digest dietary components such as plant cell walls but also to detoxify a range of plant defense compounds (Ratzka et al., 2002; Whiteman and Jander, 2010). Our previous study of the *P. xylostella* genome reveals the mechanism of detoxification of some plant defense compounds, particularly glucosinolates (You et al., 2013). Other compounds including phenolics are, however, abundant in Brassicacea species (Cartea et al., 2010) and comprise an important part of the plant's defense against herbivores. Phenolics are harmful to insects as toxic or anti-digestive compounds (Bi and Felton, 1995; Duffey and Stout, 1996; Mazid et al., 2011). It has been suggested that microorganisms can degrade phenol and catechol by both aerobic and anaerobic pathways (Basha et al., 2010).

**Figure 5:**
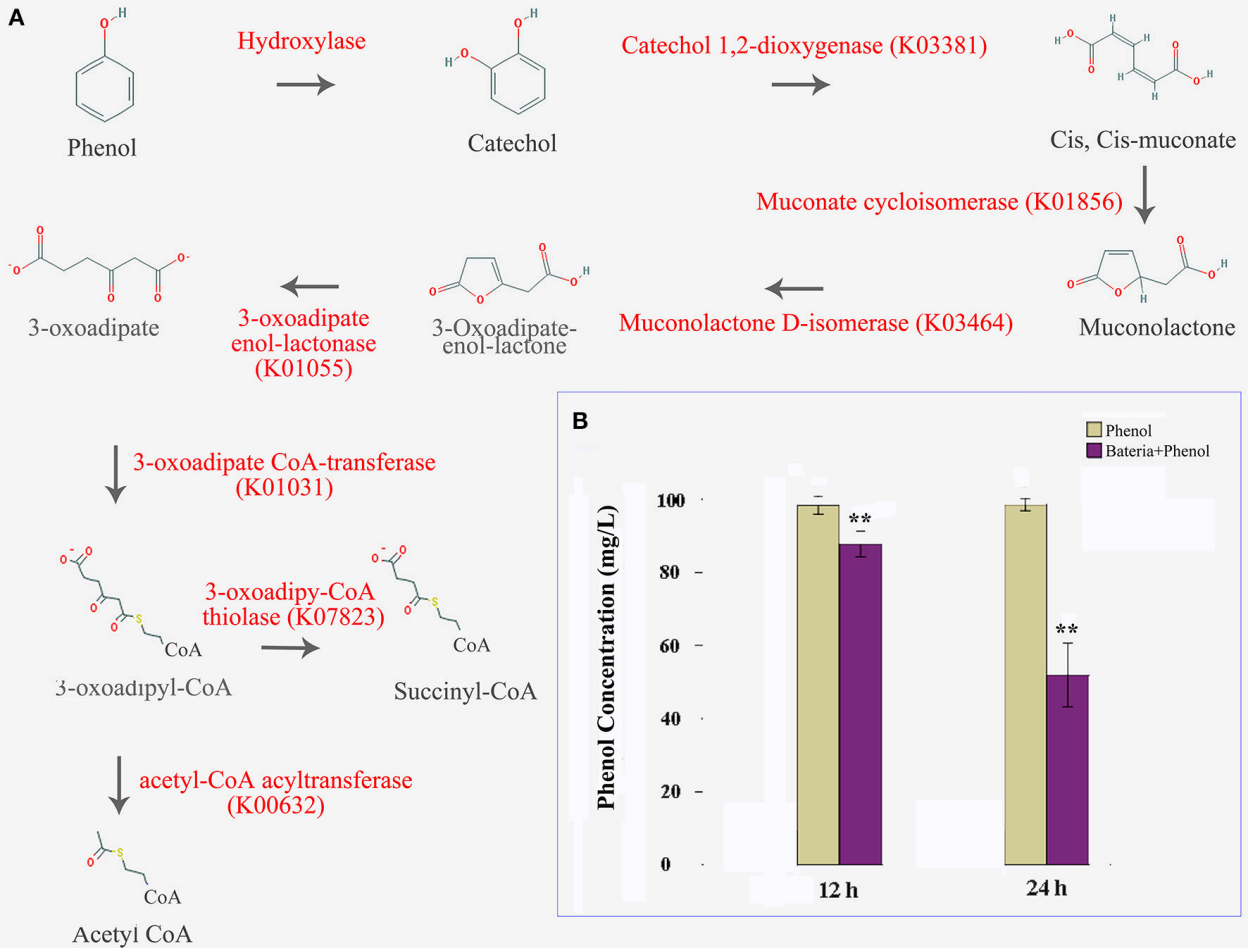
*****P. xylostella*** gut bacteria-mediated biodegradation of phenols**. Aerobe pathway of phenol biodegradation, enzymes beside the arrow lines are identified from the metagenome **(A)**, and degradation efficiency at 12 and 24 h after treatment with colored histograms representing different treatments **(B)**. Yellow column indicated that the phenol media without the presence of bacteria, and the purple indicated that the phenol media with the presence of *P. xylostella* gut bacteria, the stars ^**^ over the columns within a graph denote significant differences (*p* < 0.01) between the different treatments.

In this study, we were able to identify the entire aerobic pathway for catechol degradation in the *P. xylostella* gut bacteria symbiont metagenome, including catechol 1,2-dioxygenase, muconate cycloisomerase, muconolactone D-isomerase, 3-oxoadipate enol-lactonase, 3-oxoadipate CoA-transferase, acetyl-CoA acyltransferase, 3-oxoadipyl-CoA thiolase, and 3-oxoadipate enol-lactonase/4-carboxymuconolactone decarboxylase (Figure 5A, Supplementary Table 13). All of these genes were encoded by *Enterobacter* (especially *E. asburiae* and *E. cloacae*). We believe that these two species confer protection to the host insect against certain plant defense compounds of Brassica plants. Further functional analytical experiments indicated that the *P. xylostella* gut bacteria could degrade the phenol significantly *in vitro* (Figure 5B).

Benzoate is a simple aromatic carboxylic acid that occurs naturally in many plants and serves as an intermediary for many plant secondary metabolites (Qualley et al., 2012). A series of enzymes participating in benzoate degradation were identified in the metagenome including benzoate 1,2-dioxygenase alpha subunit, benzoate 1,2-dioxygenase beta subunit, and benzoate-CoA ligase (Supplementary Figure 10, Supplementary Table 14). A major pathway for benzoate degradation had catechol as an intermediate step (Supplementary Figure 10). In addition, enzymes participating in the degradation of aromatic compounds, such as 2-[hydroxy(phenyl)methyl]-succinyl-CoA dehydrogenase BbsD subunit for toluene biodegradation, p-cumic alcohol dehydrogenase, p-cumate dioxygenase, and p-cumic aldehyde dehydrogenase for xylene, citronellol/citronellal dehydrogenase for geraniol, salicylate hydroxylase, and 2,6-dioxo-6-phenylhexa-3-enoate hydrolase for dioxin, and N-ethylmaleimide reductase, nitroreductase/dihydropteridine reductase, and nitroreductase for nitrotoluene, were detected in the *P. xylostella* gut microbiota (Supplementary Table 14). These aromatic compounds may affect the gut environment and modulate the microbiota (Lee et al., 2006; Xu et al., 2013). Thus, we speculate that the abundance of aromatic compound degradation enzymes in the *P. xylostella* gut microbiota may have led to co-benefits for *P. xylostella* and its gut microbiota.

Some plant phenolics, such as phenol, catechol, chlorogenic acid, and caffeic acid, are directly or indirectly toxic to herbivores by stimulating DNA degradation via the induction of reactive oxygen species (ROS) when they are oxidized (Moran et al., 1997; Schweigert et al., 2000, 2001). Plants wounded by insects can increase production of ROS, resulting in oxidative damage to the herbivore midgut (Bi and Felton, 1995). ROS have been shown to be toxic to larvae of several species of lepidopteran and their expressions must be carefully controlled (Ahmad et al., 1987; Ahmad and Pardini, 1990; Duffey and Stout, 1996). The concentration and conversion of ROS to nontoxic compounds can be regulated by superoxide dismutases (SODs), peroxidase, and catalases (Bandyopadhyay et al., 1999; DeJong et al., 2007; Mittapalli et al., 2007). Our study identified 11 SOD genes, 14 catalase genes and 29 peroxidase genes in *P. xylostella* gut bacterial symbionts (Supplementary Table 15). These genes were encoded by *C. maltaromaticum* and *Enterobacter* spp., especially *E. asburiae* and *E. cloacae*. These abundant species participate in the detoxification of ROS and can provide strong protection for the host insect. In addition, *Enterobacter* spp., especially *E. asburiae* and *E. cloacae*, also contained genes encoding carboxylesterase (COE) and glutathione S-transferase (GST) (Supplementary Table 16). These enzymes induce xenobiotic detoxification (Li et al., 2007; You et al., 2013). The GST family of *Drosophila melanogaster* can be induced under phenol exposure (Shen et al., 2003). Our results support the hypothesis that these microbiota enzymes have a potential ability to enhance resistance of *P. xylostella* gut to ROS.

### Amino acid synthesis in the gut microbiota of *P. xylostella*

**Figure 6:**
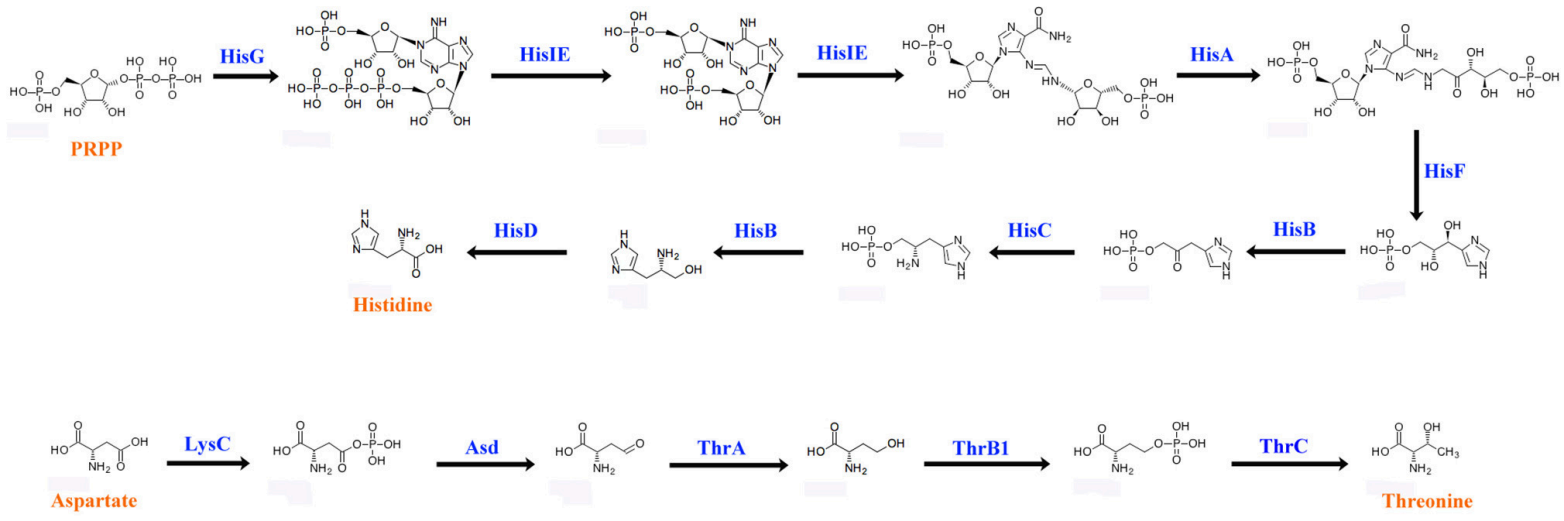
**Biosynthetic pathways of histidine and threonine by ***P. xylostella*** gut bacteria**. The blue words above the arrow lines representing the enzymes of amino acid biosynthesis that identified from the metagenome.

Animals cannot synthesize some essential amino acids and must therefore obtain them from food (Hansen and Moran, 2011). To compensate for host deficiency, host symbiont cooperation in producing essential amino acids is a common phenomenon in phloem-feeding insects (Baumann, 2005), for example, symbiotic bacteria of the pea aphid synthesize arginine (Hansen and Moran, 2011). Based on *P. xylostella* genome database (Tang et al., 2014), *P. xylostella* lacks the entire pathway for the synthesis of histidine (His) from phosphoribosyl pyrophosphate (PRPP) as well as the pathway for the synthesis of threonine (Thr) from aspartate (Asp). We found that the *P. xylostella* gut bacteria metagenome possessed the whole pathway for synthesis of His and Thr (Figure 6). Our results are significant as they suggest that *P. xylostella*-symbiont cooperates with its host to produce these lacking amino acids, thus having an important impact on host nutrition. The genes participating in the synthesis of those amino acids were found mainly in *E. asburiae, E. cloacae*, and *C. maltaromaticum* (Supplementary Table 17). Whilst the symbiotic bacteria of pea aphid are intracellular (Hansen and Moran, 2011), in *P. xylostella*, it is unclear whether these synthesized amino acids are secreted into the intestine for direct use by the host or are retained by the symbiotic bacteria and available to the host only on decomposition of the bacteria. Further research is needed to identify these pathways; nevertheless, our study opens an avenue for the study of nutrition interaction between *P. xylostella* and its gut bacteria.

## Conclusions

Our study based on metagenomic analysis of *P. xylostella* gut microbiota using next-generation sequencing provides a detailed profile of taxonomic and functional information regarding the *P. xylostella* gut microbiota. We identified a series of enzymes encoded by bacteria that participate in food digestion, nutrition, and the detoxification of plant defense compounds, especially of phenolic and ROS detoxification. The results suggest that while the bacterial community in the *P. xylostella* gut is complex, the main species participating in those functions are few, with *E. asburiae, E. cloacae*, and *C. maltaromaticum* being the three most abundant species. Comparative analysis of the profiles of plant tissue degradation enzymes detected in different insects has revealed that the abundance and diversity of the digestion enzymes are linked to the diet of these insects. This highlights the complexity of the possible co-evolution of gut microbiota and the host insect to cope with the challenges posed by its diet. Although, there were some differences of the gut microbiota composition between larvae and pupae/adult in *P. xylostella* gut, the extent was much lower compared with some other lepidopteran insects, such as *Heliconius erato* (Hammer et al., 2014), *Heliothis virescens* (Staudacher et al., 2016), and *Spodoptera littoralis* (Chen et al., 2016). In these three lepidopteran insects, the gut microbiota greatly varies across different developmental stages. In contrast, we found that *P. xylostella* gut microbiota were much consistent through life stages and further the functions classified by KEGG were identical. This may be a unique phenomenon to *P. xylostella* leading to a new question: does the same set of bacteria perform different functions during host development? Future studies should include metatranscriptomics and metaproteomics to validate this phenomenon and thus clarify the expressions of functional genes of the gut *in vivo*.

Our research, combined with the previous *P. xylostella* genome studies, indicates that its gut microbe complements its own genome allowing the resulting holobiont to function as an evolutionarily adaptive herbivore and assuming the status as one of the world's most serious pests. Accordingly, information on the gut microbiota, particularly the bacterial species and functional groups that play a particularly important role for the host's success, offers scope for future studies to develop new pest management approaches that exploit novel targets of chemical, genetic and biological control for this and other insect pests.

## Author contributions

XX and MY designed the research; XX, DZ, JL, HL, FS, and YL performed the research; XX, HZ, BQ, and YW analyzed the data; MY and XX contributed reagents/materials/analysis tools; XX, MY, GG, and LV wrote the paper; and MY, XX, GG, and LV revised the paper.

## Funding

This work, including the efforts of MY was funded by the projects of the National Natural Science Foundation of China (Nos. 31230061 and 31320103922), XX was supported by the project of the National Natural Science Foundation of China (Nos. 31501639).

### Conflict of interest statement

The authors declare that the research was conducted in the absence of any commercial or financial relationships that could be construed as a potential conflict of interest.
