# Supplementary figures and images for: Metagenomic Sequencing of Diamondback Moth Gut Microbiome Unveils Key Holobiont Adaptations for Herbivory
Source: Front Microbiol. 2017 Apr 26;8:663. doi: 10.3389/fmicb.2017.00663 (PMC5405146; doi:10.3389/fmicb.2017.00663)

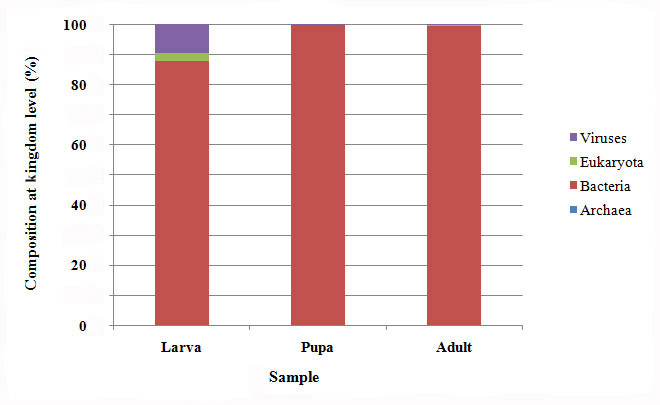

Supplement: Supplementary file 1 [file Presentation1.zip › Supplementary Figures/Supplementary Figure 1.jpg]

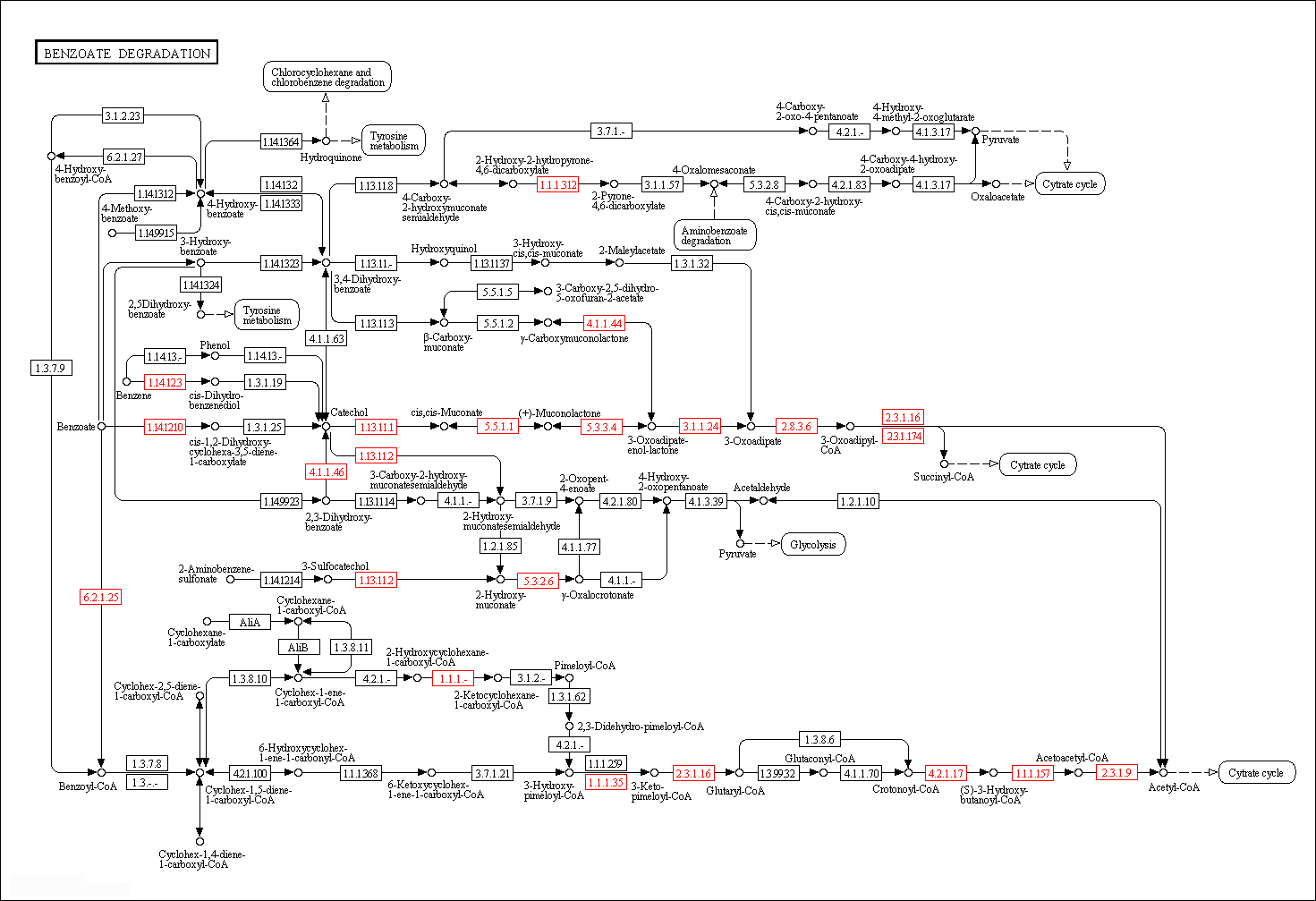

Supplement: Supplementary file 1 [file Presentation1.zip › Supplementary Figures/Supplementary Figure 10.png]

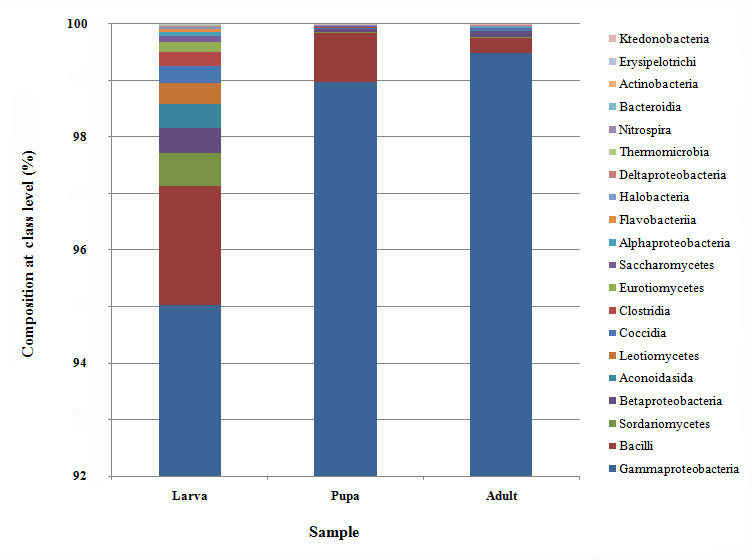

Supplement: Supplementary file 1 [file Presentation1.zip › Supplementary Figures/Supplementary Figure 2.jpg]

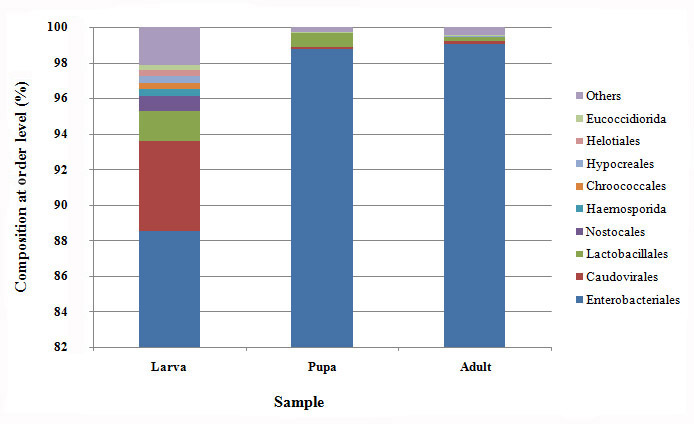

Supplement: Supplementary file 1 [file Presentation1.zip › Supplementary Figures/Supplementary Figure 3.jpg]

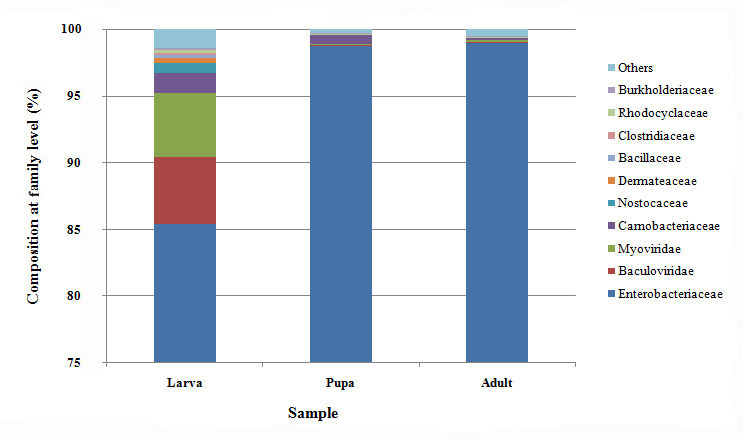

Supplement: Supplementary file 1 [file Presentation1.zip › Supplementary Figures/Supplementary Figure 4.jpg]

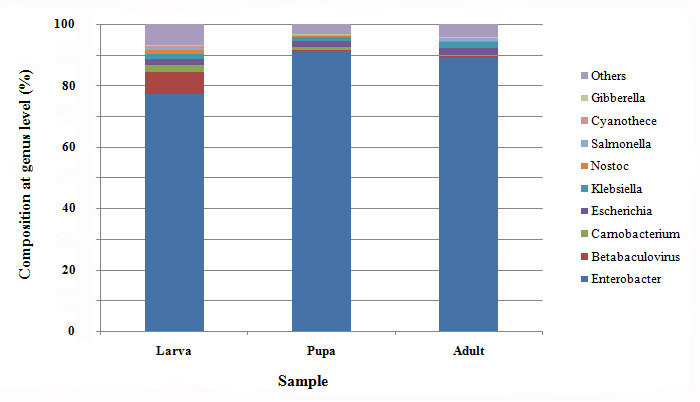

Supplement: Supplementary file 1 [file Presentation1.zip › Supplementary Figures/Supplementary Figure 5.jpg]

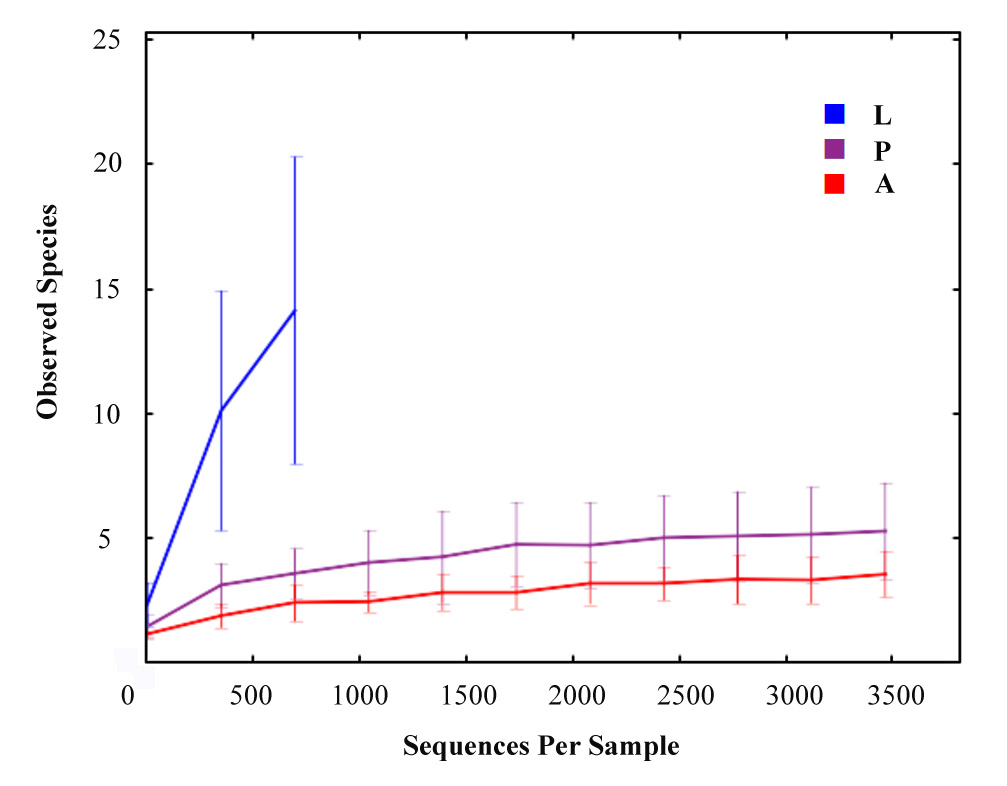

Supplement: Supplementary file 1 [file Presentation1.zip › Supplementary Figures/Supplementary Figure 6.jpg]

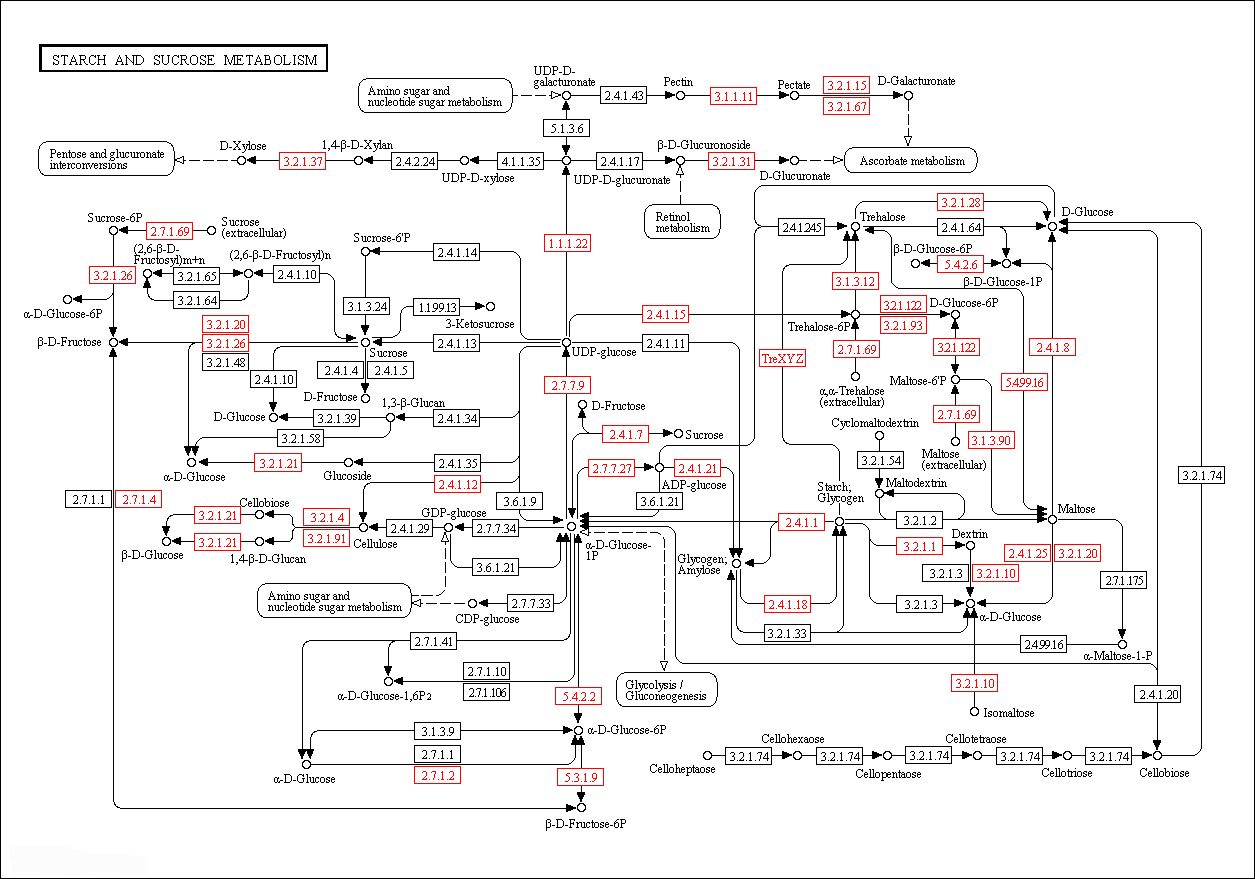

Supplement: Supplementary file 1 [file Presentation1.zip › Supplementary Figures/Supplementary Figure 8.png]

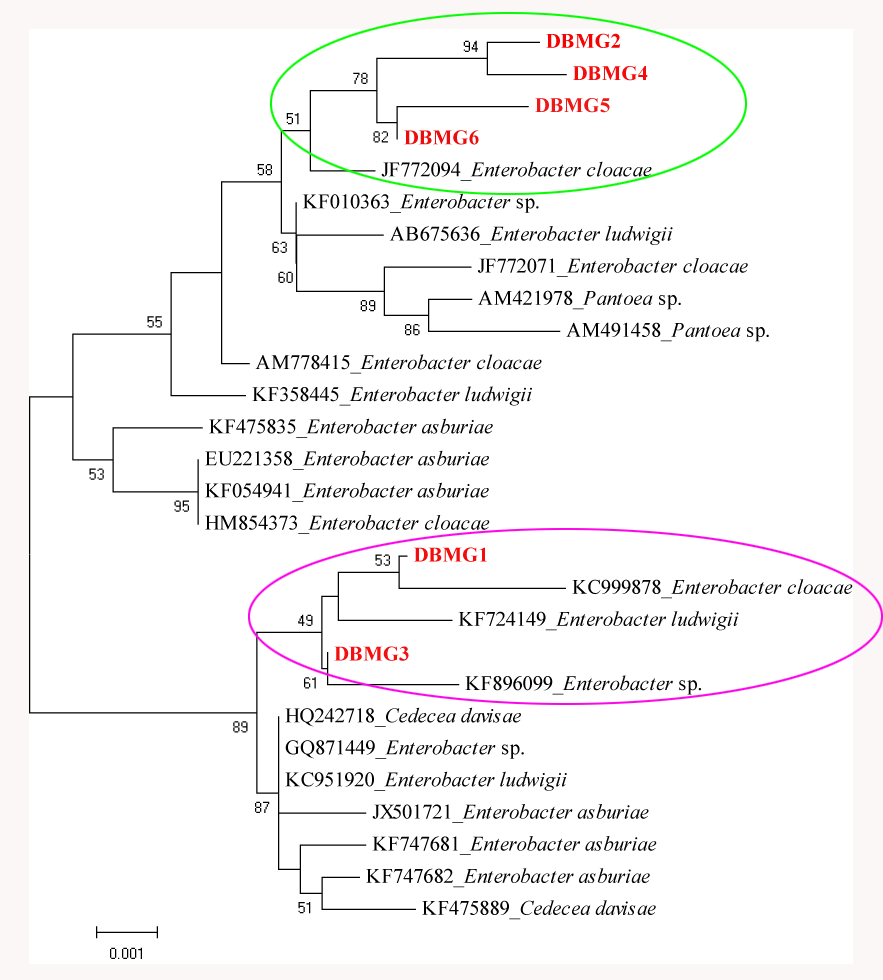

Supplement: Supplementary file 1 [file Presentation1.zip › Supplementary Figures/Supplementary Figure 9.png]
