# Supplementary material for: Metagenomic Sequencing of Diamondback Moth Gut Microbiome Unveils Key Holobiont Adaptations for Herbivory
Source: Front Microbiol. 2017 Apr 26;8:663. doi: 10.3389/fmicb.2017.00663 (PMC5405146; doi:10.3389/fmicb.2017.00663)
Supplement: Supplementary file 3 [file DataSheet1.docx]

**Supplementary Figure Legends**

**Supplementary Figure 1. Proportional composition of microbiota in the *P. xylostella* gut at the kingdom level**.

**Supplementary Figure 2. Proportional composition of microbiota in the *P. xylostella* gut at the class level**.

**Supplementary Figure 3. Proportional composition of microbiota in the *P. xylostella* gut at the order level.**

**Supplementary Figure 4. Proportional composition of microbiota in the *P. xylostella* gut at the family level.**

**Supplementary Figure 5. Proportional composition of microbiota in the *P. xylostella* gut at the genus level.**

**Supplementary Figure 6. The rarefaction curves of *P. xylostella* gut microbiota generated by 454 pyrosequencing. L: Larva, P: Pupa and A: Adult.**

**Supplementary Figure 7. Projection of the KEGG pathways of the *P. xylostella* genome and the gut microbiota metagenome analyzed by iPath2.** The blue pathways are specific in the *P. xylostella* genome, the red pathways are specific in the metagenome, and the green lines are pathways in both genomes.

**Supplementary Figure 8. The starch- and sucrose-metabolic enzymes (red boxes) identified in the *P. xylostella* gut microbiota.**

**Supplementary Figure 9. Phylogenetic analysis of cellulose-, xylan- and pectin-biodegrading bacteria in *P. xylostella* gut.** Sequences isolated from the 4th-instar larval gut of *P. xylostella* are marked with red. All positions containing gaps and missing data are eliminated. The bootstrap test (1000 replicates) is shown next to the branches.

**Supplementary Figure 10. The benzoate-metabolic enzymes (red boxes) identified in the *P. xylostella* gut microbiota.**
