# Supplementary material for: Metagenomic Sequencing of Diamondback Moth Gut Microbiome Unveils Key Holobiont Adaptations for Herbivory
Source: Front Microbiol. 2017 Apr 26;8:663. doi: 10.3389/fmicb.2017.00663 (PMC5405146; doi:10.3389/fmicb.2017.00663)
Supplement: Supplementary file 4 [file DataSheet2.docx]

**Supplementary Table Legends**

**Supplementary Table 1.** Numerical data of the *P. xylostella* gut microbiota metagenome.

**Supplementary Table 2.** SOAP *de novo* assembling of the *P. xylostella* gut microbiota metagenome.

**Supplementary Table 3.** Gene prediction based on the *P. xylostella* gut microbiota metagenome.

**Supplementary Tables 4.** Taxonomic profile of P. xylostella gut microbiota based on the metagenomics analysis.

**Supplementary Table 5.** Numerical data of the *P. xylostella* gut microbiota sequenced by 454 pyrosequencing.

**Supplementary Tables 6.** Species profiling and OTU information of P. xylostella gut microbiota studied by 454 pyrosequencing.

**Supplementary Table 7.** Alpha diversity of microbiota in *P. xylostella* gut sequenced by 454 pyrosequencing.

**Supplementary Table 8.** Taxonomic distribution of assigned reads of the *P. xylostella* gut microbiota sequenced by 454 pyrosequencing.

**Supplementary Tables 9.** Carbohydrate breakdown enzymes detected in the P. xylostella gut microbiota.

**Supplementary Table 10.** Family-based numerical comparison of the genes encoding carbohydrate-active enzymes in different species metagenomes.

**Supplementary Table 11.** Lignocellulose biodegradation genes in the *P. xylostella* gut microbiota metagenome.

**Supplementary Table 12.** Sequence identity matrix of 16S rRNA of cellulose-, xylan- and pectin- biodegrading bacteria identified from the *P. xylostella* gut.

**Supplementary Table 13.** Catechol-biodegrading genes in the *P. xylostella* gut microbiota.

**Supplementary Table 14.** Gene families participated in detoxification of benzoate and aromatic compounds in the *P. xylostella* gut microbiota.

**Supplementary Table 15.** Genes participated in ROS detoxification in the *P. xylostella* gut microbiota.

**Supplementary Table 16.** The COE and GST genes in the *P. xylostella* gut microbiota.

**Supplementary Table 17.** The amino acid biosynthesizing genes in the *P. xylostella* gut microbiota.
